# Le rhinosclérome une infection chronique rare des fosses nasales

**DOI:** 10.11604/pamj.2018.31.247.14104

**Published:** 2018-12-26

**Authors:** Souha Kallel, Abdel Mounem Ghorbel

**Affiliations:** 1 Service ORL et Chirurgie Cervico-Faciale, CHU Habib Bourguiba, 3029 Sfax, Tunisie

**Keywords:** Rhinosclérome, fosse nasale, chirurgie, Rhinoscleroma, nasal fossa, surgery

## Abstract

We report the case of a 80-year old hypertensive female patient, presenting with bilateral nasal obstruction not improved by symptomatic treatment. The examination of the nasal cavity showed pseudotumoral hypervascularized granulomatous bleeding lesion on both sides of the anterior portion of the nasal septum (A). The remainder of the septal mucosa showed granulomatous appearance. CT scan of the facial bones showed tissue lesion of the nasal cavities on both sides of the septum which was moderately enhancing after injection of iodinated contrast medium (ICM), without bone lysis (B). Biopsy showed rhinoscleroma. The patient received combination therapy including cyclin associated with cotrimoxazole without disappearance of the nasal lesion. Endonasal excision of the septal mass was performed. Histological examination confirmed the diagnosis of rhinoscleroma. The patient underwent ciprofloxacin therapy for 1 month. Patient evolution was good with nasal clearance at 1-year follow up. Rhinoscleroma is a granulomatous infection of the nasal cavities due to enteric bacterium belonging to the Klebsiella family (Klebsiella rhinoscleromatis). Treatment is essentially medical. Surgery is used to remove obstructive pseudotumoral lesions resistant to medical treatment.

## Image en médecine

Il s'agit d'une patiente âgée de 80 ans, hypertendue, consultant pour une obstruction nasale bilatérale non améliorée par le traitement symptomatique. L'examen des fosses nasales a montré une lésion granulomateuse pseudotumorale hyper vascularisée et saignante au contact de part et d'autre de la partie antérieure de la cloison nasale (A) avec un aspect granulomateux du reste de la muqueuse septale. Le scanner du massif facial a montré une lésion tissulaire des fosses nasales de part et d'autre du septum se rehaussant modérément après injection des produits de contraste (PDC) sans lyse osseuse en regard (B). La biopsie a conclu au diagnostic de rhinosclérome. Une bithérapie incluant une cycline avec Cotrimoxazole a été prescrite mais sans disparition de la lésion nasale. D'où le recours à une chirurgie endonasale avec exérèse de la masse septale. L'examen histologique définitif a confirmé le diagnostic de rhinosclérome. La patiente a été mise sous ciprofloxacine pendant 1 mois. L'évolution était bonne avec désobstruction des fosses nasales avec un recul de 1 an. En conclusion, le rhinosclérome est une infection granulomateuse des fosses nasales due à une entérobactérie de la famille Klebsiella (*Klebsiella Rhinoscleromatis*). Le traitement est essentiellement médical. La chirurgie s'adresse aux lésions pseudotumorales obstructives résistantes au traitement médical.

**Figure 1:**
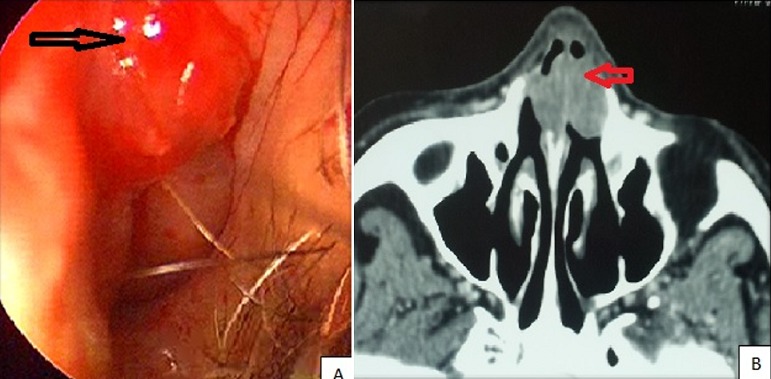
présentation du rhinosclérome: A) lésion pseudotumorale hyper vascularisée et saignante au contact de la partie antérieure de la cloison nasale; B) TDM du massif facial en coupe axiale qui montre un processus expansif bilobé des deux fosses nasales centré sur la partie antérieure du septum

